# Five Years After the Onset of the COVID-19 Pandemic: Changing Epidemiology of SARS-CoV-2 and Seasonal Respiratory Viruses in Hospitalized Adults

**DOI:** 10.3390/v18101057

**Published:** 2026-09-24

**Authors:** Mustafa Usanmaz, Özgür Günal, Burcu Özdemir, Esra Arslan Aksu, Savaş Gegin, Muhammed Abdullah, İkra Nur Gergin, Furkan Ata, Merve Meryem Öztürk, Meltem Karslıoğlu, Esmeray Mutlu Yılmaz, Ayşe Şenbaba Kara, Ayşe Banu Ceviz, Levent Özdemir

**Affiliations:** 1 Department of Infectious Diseases, Faculty of Medicine, Samsun University, Canik Campus, 55080 Samsun, Türkiye; mustafa.usanmaz@samsun.edu.tr (M.U.); ozgurgop@yahoo.com (Ö.G.); mervemeryemozturk@gmail.com (M.M.Ö.); drmeltemkarslioglu@gmail.com (M.K.); ayse-senbaba@hotmail.com (A.Ş.K.); abanuceviz@gmail.com (A.B.C.); 2 Department of Pulmonology, Faculty of Medicine, Samsun University, Canik Campus, 55080 Samsun, Türkiye; eydu2408@gmail.com (B.Ö.); dresraarslan07@gmail.com (E.A.A.); mokhangef2021@gmail.com (M.A.); ikragergin@gmail.com (İ.N.G.); furkan202furkan@hotmail.com (F.A.); 3 Department of Pulmonology, Samsun City Hospital, 55080 Samsun, Türkiye; geginn@hotmail.com; 4 Department of Infectious Diseases, Samsun City Hospital, 55080 Samsun, Türkiye; emutlu55@gmail.com

**Keywords:** SARS-CoV-2, influenza, respiratory syncytial virus, respiratory viruses, viral co-detection, hospitalized adults, post-pandemic surveillance

## Abstract

Pandemic non-pharmaceutical interventions disrupted seasonal respiratory virus circulation, but longer-term pathogen-specific patterns in hospitalized adults remain incompletely defined. We evaluated detections across three nonconsecutive respiratory seasons from early relaxation of pandemic measures to five years after pandemic onset. This retrospective, observational, single-center cohort study included 2249 adults hospitalized with pneumonia, chronic obstructive pulmonary disease exacerbation, or asthma exacerbation who underwent multiplex polymerase chain reaction testing in September 2021–May 2022, September 2022–May 2023, or September 2025–May 2026. Influenza A positivity peaked in the second period (12.6%) and decreased to 6.8% in the third (*p* < 0.001). SARS-CoV-2 likewise peaked in the second period (9.2%) and declined to 1.5% in the third (*p* < 0.001). Respiratory syncytial virus increased from 0.5% in the first period to 4.9% and 3.8% in the second and third periods, respectively (*p* < 0.001). Positivity for other respiratory viruses did not differ (16.0%, 16.9%, and 19.1%; *p* = 0.308). Viral co-detection was highest in the second period (6.1% vs. 2.6% and 2.8%; *p* < 0.001). Respiratory virus epidemiology remained dynamic five years after pandemic onset, supporting sustained multi-pathogen surveillance.

## 1. Introduction

Lower respiratory tract infections are among the leading causes of hospitalization, morbidity, and mortality in adults worldwide [1]. Viral pathogens are important etiologic agents of lower respiratory tract infection. Influenza viruses, respiratory syncytial virus (RSV), rhinoviruses, seasonal human coronaviruses, and severe acute respiratory syndrome coronavirus 2 (SARS-CoV-2) are major causes of severe acute respiratory infection in adults [2].

The COVID-19 pandemic not only introduced SARS-CoV-2 as a new respiratory pathogen but also substantially altered the circulation of other common respiratory viruses [3]. Mask use, physical distancing, travel restrictions, and other non-pharmaceutical public health measures were associated with marked reductions in the circulation and diversity of seasonal respiratory viruses [4,5,6,7]. After these measures were relaxed, respiratory viruses re-emerged; however, a simultaneous return to pre-pandemic seasonal patterns was not observed across all regions or viral pathogens. Some viruses produced earlier-than-expected epidemics, whereas others showed substantial changes in the timing or intensity of circulation [8].

In particular, RSV showed shifts in its seasonal circulation following the relaxation of pandemic-related public health measures [8].

Although numerous studies have described the first respiratory virus seasons after the pandemic, many focused on the early post-pandemic period or evaluated only short-term epidemiologic changes [8]. More recent surveillance studies have assessed multiple respiratory viruses concurrently, but comparative data on pathogen-specific patterns during later years after pandemic onset remain limited [9]. Characterizing these changes is important for surveillance strategies, diagnostic planning, vaccination policies, and public health preparedness.

This study aimed to evaluate temporal changes in respiratory viral detections among adults hospitalized during three respiratory virus seasons after the onset of the COVID-19 pandemic. We compared the distribution of SARS-CoV-2 and seasonal respiratory viruses and the frequency of viral co-detection across study periods. We also described the distribution of viral pathogens according to the primary admission diagnosis.

## 2. Materials and Methods

### 2.1. Study Design and Setting

This retrospective, observational, single-center cohort study was conducted in the pulmonology and infectious diseases clinics of Samsun City Hospital, Samsun, Türkiye. The study included adults hospitalized during three respiratory virus seasons: September 2021–May 2022, September 2022–May 2023, and September 2025–May 2026. Eligible patients had a primary admission diagnosis of pneumonia, acute exacerbation of chronic obstructive pulmonary disease (COPD), or asthma exacerbation and underwent multiplex polymerase chain reaction (PCR) testing of a respiratory specimen at admission.

### 2.2. Participants and Data Sources

Patients aged ≥18 years were eligible if they were hospitalized in one of the prespecified periods, underwent a respiratory viral panel at admission, and had pneumonia, COPD exacerbation, or asthma exacerbation as the primary admission diagnosis. The three diagnostic groups were mutually exclusive. Patients aged <18 years, those with missing or unavailable PCR results, and repeat specimens obtained during the same hospitalization were excluded; when more than one specimen was obtained during a hospitalization, only the first specimen was retained.

Demographic and clinical data were obtained from the FONET Hospital Information Management System, medical records, and physician progress notes. Age, sex as recorded in the electronic record, primary admission diagnosis, and comorbidities were collected. Respiratory viral PCR results were obtained from the Republic of Türkiye Ministry of Health Public Health Management Information System.

### 2.3. Study Periods

The three periods were defined a priori to represent different phases of public health practice after pandemic onset. Period 1 (September 2021–May 2022) represented the early relaxation phase, when most restrictions had been eased but mask requirements remained in selected settings. Period 2 (September 2022–May 2023) represented the first respiratory season after removal of the general mask mandate. Period 3 (September 2025–May 2026) represented the respiratory season approximately five years after pandemic onset. The nonconsecutive periods were selected to compare early and later changes in respiratory virus circulation.

### 2.4. Respiratory Specimens and Multiplex PCR

Respiratory specimens comprised nasopharyngeal swabs and/or lower respiratory tract specimens (sputum, tracheal aspirate, or bronchoalveolar lavage). The multiplex PCR panel included SARS-CoV-2; influenza A; influenza A subtypes A (H1N1) and A (H3N2); influenza B; adenovirus; seasonal human coronaviruses HKU1, 229E, NL63, and OC43; enterovirus; rhinovirus; human bocavirus; human metapneumovirus; human parainfluenza virus types 1–4; RSV A/B; and parechovirus. Enterovirus and rhinovirus were analyzed as separate targets by the assay but were combined as Enterovirus/Rhinovirus in the overall virus-distribution analyses.

### 2.5. Outcomes and Definitions

Viral co-detection was defined as the simultaneous detection of two or more viral targets in the same clinical specimen. The primary outcome was the difference in the distribution of respiratory viral detections across the three study periods. The secondary outcome was the difference in viral co-detection frequency across periods. The distribution of viral pathogens by primary admission diagnosis was evaluated descriptively. The category ‘other respiratory viruses’ comprised all panel viruses other than influenza A, influenza B, and SARS-CoV-2. Because more than one target could be detected in a specimen, virus-specific categories were not mutually exclusive.

### 2.6. Ethics

The study was conducted in accordance with the Declaration of Helsinki and was approved by the Samsun University Non-Interventional Clinical Research Ethics Committee (approval date: 1 July 2026; decision No. 2026/12/6).

### 2.7. Statistical Analysis

Statistical analyses were performed using IBM SPSS Statistics for Windows, version 23.0 (IBM Corp., Armonk, NY, USA). The normality of continuous variables was assessed using the Kolmogorov–Smirnov test, and the data were found to be normally distributed. Continuous variables are summarized as mean ± standard deviation (SD), and categorical variables as number (n) and percentage (%). Associations between study period and respiratory viral detection were evaluated using Pearson’s chi-square test. Fisher’s exact test was used when expected cell counts were <5. For variables with a statistically significant overall difference across the three study periods, pairwise post-hoc comparisons between periods were performed, with Bonferroni correction for multiple comparisons. Viral distributions by primary admission diagnosis are presented descriptively. All tests were two-sided, and *p* < 0.05 was considered statistically significant.

### 2.8. Generative AI Use

During the preparation of this manuscript, ChatGPT (GPT-5.6 Luna; OpenAI, San Francisco, CA, USA; accessed August 2026) was used for English translation, language editing, and journal-format restructuring. All AI-assisted output was reviewed and edited by the authors, who take full responsibility for the final content of the manuscript.

## 3. Results

### 3.1. Patient Characteristics

A total of 2249 patients were included: 424 (18.9%) in Period 1, 911 (40.5%) in Period 2, and 914 (40.6%) in Period 3. The mean age was 67.4 ± 21.5 years; 1397 patients (62.1%) were male and 852 (37.9%) were female. At least one comorbidity was present in 1997 patients (88.8%). COPD exacerbation was the most frequent admission diagnosis (*n* = 1024, 45.5%), followed by pneumonia (*n* = 757, 33.7%) and asthma exacerbation (*n* = 468, 20.8%). Patient characteristics are summarized in Table 1.

### 3.2. Viral Distribution by Admission Diagnosis

The distribution of viral detections according to admission diagnosis is shown in Table 2. Influenza A positivity was 8.6%, 9.8%, and 10.7% among patients with COPD exacerbation, pneumonia, and asthma exacerbation, respectively, while SARS-CoV-2 positivity was 4.0%, 7.7%, and 3.6%, respectively. Other respiratory viruses were also detected across all three diagnostic groups, with detailed virus-specific frequencies presented in Table 2.

### 3.3. Temporal Changes in Viral Detections

Viral detections by study period are shown in Table 3. Influenza A, A (H1N1), SARS-CoV-2, and RSV A/B differed significantly across periods. Influenza A positivity was highest in Period 2 (12.6%) and declined to 6.8% in Period 3 (*p* < 0.001). A (H1N1) positivity similarly increased to 11.0% in Period 2 and decreased to 1.5% in Period 3 (*p* < 0.001). SARS-CoV-2 positivity peaked at 9.2% in Period 2 and declined to 1.5% in Period 3 (*p* < 0.001). RSV A/B increased from 0.5% in Period 1 to 4.9% in Period 2 and remained at 3.8% in Period 3 (*p* < 0.001). Overall positivity for other respiratory viruses did not differ significantly (*p* = 0.308). The principal temporal patterns are illustrated in Figure 1.

### 3.4. Viral Co-Detection

Viral co-detection was observed in 93 patients (4.1%). The frequency was highest in Period 2 (6.1%), compared with 2.6% in Period 1 and 2.8% in Period 3 (*p* < 0.001; Table 4). The distribution of viral combinations among patients with co-detection according to the three study periods is presented in Table 5. Each patient was counted once according to the complete viral detection pattern, with patients in whom three or more viruses were detected grouped separately.

## 4. Discussion

This study demonstrates pathogen-specific temporal patterns in respiratory virus detections among hospitalized adults after the onset of the COVID-19 pandemic. SARS-CoV-2 positivity increased in the second period and then declined markedly by the season approximately five years after pandemic onset. Influenza A showed a similar peak in the second period followed by a decline in the third period. In contrast, RSV was detected infrequently in the first period, increased substantially in the second period, and continued to circulate in the third period. Overall positivity for other respiratory viruses did not change significantly. Viral co-detection also peaked in the second period and subsequently decreased. These findings indicate that post-pandemic respiratory virus epidemiology has not followed a uniform trajectory but has remained pathogen-specific and temporally dynamic.

Coronary artery disease was present in 21.7% of our cohort. In a comparable hospital-based study of adults hospitalized with Influenza A, SARS-CoV-2, or RSV infection, coronary artery disease was reported in 28.6%, 25.3%, and 36.4% of patients, respectively [10]. The prevalence observed in our cohort may partly reflect the advanced age of the study population.

Adult hospital surveillance studies show that SARS-CoV-2 continued to contribute to hospitalizations during successive post-pandemic seasons. In a Spanish national hospital surveillance study covering 2021–2024, SARS-CoV-2 remained an important viral pathogen among older hospitalized adults [11]. A Turkish adult hospital cohort from 2022–2024 similarly identified SARS-CoV-2 as a major respiratory viral pathogen [2]. In the 2024–2025 European id.DRIVE surveillance study across 17 hospitals, SARS-CoV-2 was detected in 6.9% of 8730 adults hospitalized with severe acute respiratory infection [12]. Against this background, the decline in SARS-CoV-2 positivity to 1.5% in our third period represents a substantial decrease relative to the earlier periods at our center. Evaluating early and later periods within the same institutional setting provides additional real-world evidence on longer-term changes in SARS-CoV-2 detection among hospitalized adults.

Influenza circulation declined markedly during the COVID-19 pandemic and resurged after relaxation of non-pharmaceutical measures. Zhao et al. analyzed data from 92 sites and showed that common respiratory viruses re-emerged asynchronously, with influenza A returning on a different timeline from other pathogens [8]. In Hong Kong, influenza activity increased substantially after mask requirements were relaxed in March 2023 [13]. Korean national surveillance data showed that influenza activity returned to pre-pandemic levels during the 2023–2024 season and reached one of the highest levels observed in recent years during 2024–2025 [14]. In our study, influenza A positivity reached 12.6% in the first respiratory season after removal of the general mask mandate and decreased to 6.8% in the third period. A (H1N1) positivity likewise decreased from 11.0% to 1.5%. These findings support continued variability in influenza circulation after its initial post-pandemic resurgence. Accordingly, the lower Influenza A positivity observed in Period 3 may reflect interseasonal variability rather than a sustained downward trend. However, the present study was not designed to identify the factors responsible for this decline, and therefore its underlying cause cannot be determined from our data.

Substantial post-pandemic changes have also been described for RSV. RSV-NET data from the United States covering 2016–2023 demonstrated a rebound in RSV-associated adult hospitalizations during the 2022–2023 season after marked reductions during the pandemic [15]. RSV was detected in 6.4% of adults hospitalized with severe acute respiratory infection in the 2024–2025 id.DRIVE surveillance study [12]. In our cohort, RSV positivity increased from 0.5% in Period 1 to 4.9% in Period 2 and remained at 3.8% in Period 3. These findings are consistent with the reported post-pandemic resurgence and show that RSV remained relevant in hospitalized adults five years after pandemic onset.

Post-pandemic changes among other respiratory viruses have varied by pathogen. Zhao et al. reported different re-emergence timelines for rhinovirus, seasonal coronaviruses, parainfluenza viruses, adenovirus, and human metapneumovirus [8]. Differences in circulation timing and seasonality were also documented in United States surveillance during 2024–2025 [16]. Although aggregate positivity for other respiratory viruses did not differ significantly across our study periods (16.0%, 16.9%, and 19.1%; *p* = 0.308), several individual targets, including human metapneumovirus and selected seasonal coronavirus and parainfluenza virus types, showed period-specific variation. These subgroup findings should be interpreted cautiously because of low counts and multiple comparisons.

Viral co-detection rates in the post-pandemic period have varied across studies. A recent Turkish study using multiplex PCR reported viral co-detection in 2.7% of adults, with SARS-CoV-2 among the frequently identified targets [17]. Another post-pandemic study reported a viral–viral co-detection rate of 6.4%, with SARS-CoV-2, rhinovirus, and influenza viruses frequently involved [18]. In our study, viral co-detection increased from 2.6% in Period 1 to 6.1% in Period 2 and then declined to 2.8% in Period 3. Among patients with two-virus co-detection, Enterovirus + Rhinovirus, Influenza A + Influenza B, and Influenza A + SARS-CoV-2 were the most frequently observed combinations. Because PCR identifies nucleic acid rather than proving active replication or a causal contribution to disease, these results are best interpreted as co-detections.

The heterogeneity observed across viruses supports integrated multi-pathogen surveillance rather than surveillance focused on a single pathogen. Continued respiratory panel surveillance may help hospitals anticipate diagnostic demand and infection-control pressures and may inform vaccination and prevention strategies for influenza, COVID-19, and RSV.

This study has several limitations. Its retrospective, single-center design limits generalizability. The study periods were non-consecutive, which precluded an uninterrupted evaluation of temporal changes in respiratory virus circulation. Furthermore, the study was not originally designed to evaluate month-to-month changes in viral circulation; therefore, monthly temporal patterns could not be assessed. Inclusion was restricted to hospitalized adults who underwent multiplex PCR at admission, creating potential selection bias and limiting inference to tested patients rather than all hospitalized adults. Changes in clinician testing behavior or admission practices across periods could not be fully addressed. Sample sizes differed across periods, reducing precision for uncommon viral targets. Vaccination status, prior infections, and other individual factors that may influence viral epidemiology were unavailable. In addition, genomic sequencing or variant-specific analysis was not performed in SARS-CoV-2-positive samples; therefore, the distribution of SARS-CoV-2 variants could not be evaluated. Male patients predominated in the study population, and virus-specific analyses were not stratified by sex; therefore, possible sex-related differences could not be evaluated. Finally, multiple target-specific comparisons and small cell counts require cautious interpretation, and the PCR-based design cannot distinguish active infection from prolonged shedding or incidental detection.

## 5. Conclusions

In this single-center study conducted in Samsun, Türkiye, respiratory virus epidemiology among hospitalized adults continued to evolve five years after the onset of the COVID-19 pandemic. SARS-CoV-2 and influenza A detections declined after peaking in the second study period, whereas RSV remained in circulation during the later period. Viral co-detection also varied over time. These pathogen-specific patterns support sustained, integrated surveillance of multiple respiratory viruses in hospitalized adults.

## Figures and Tables

**Figure 1:**
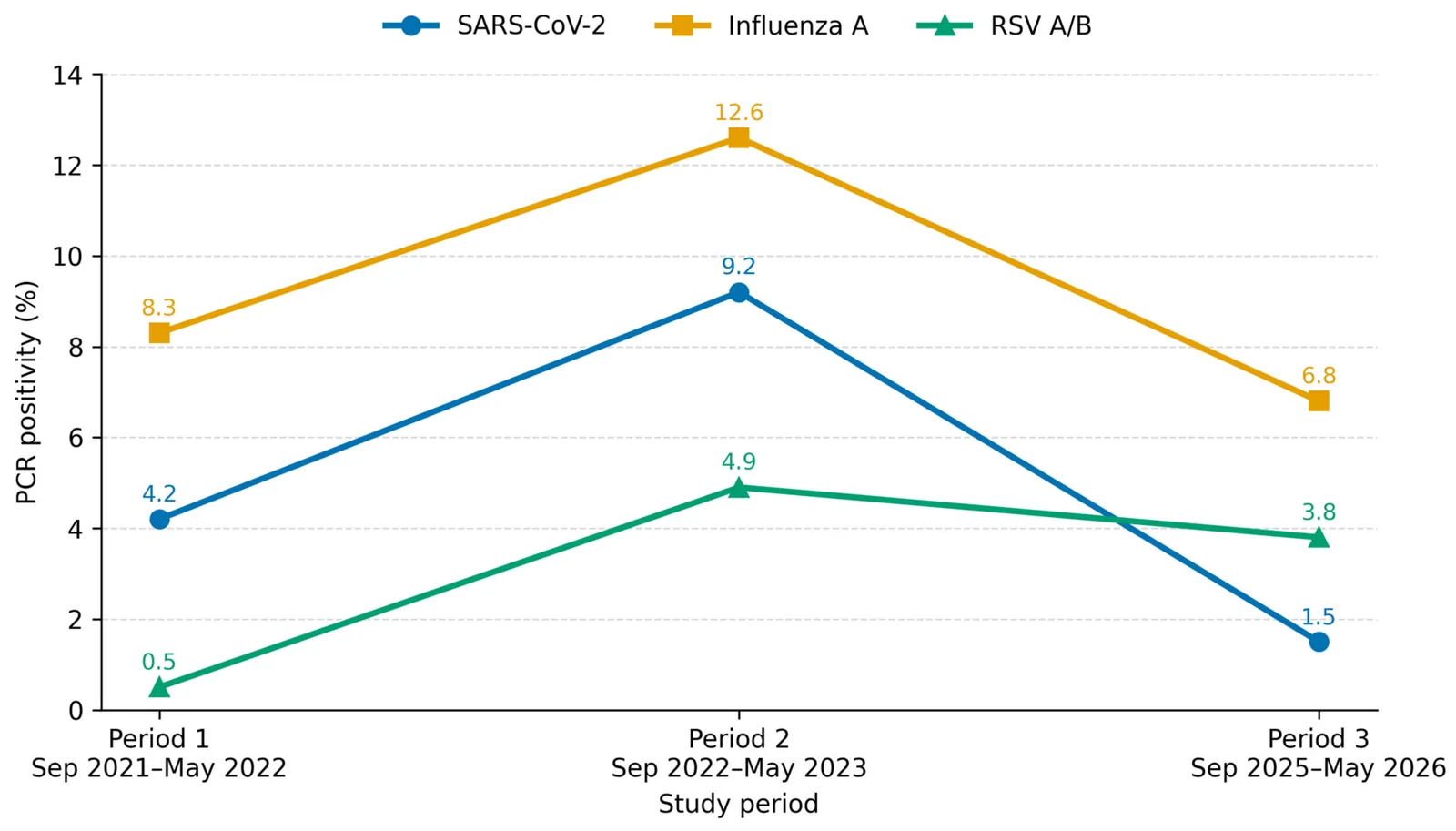
PCR positivity for SARS-CoV-2, influenza A, and RSV A/B across the three study periods. Period 1: September 2021–May 2022; Period 2: September 2022–May 2023; Period 3: September 2025–May 2026.

**Table 1:** Demographic and clinical characteristics of the study population (*n* = 2249).

Variable	Value
Study period, n (%)	
Period 1	424 (18.9)
Period 2	911 (40.5)
Period 3	914 (40.6)
Age, years, mean ± SD	67.4 ± 21.5
Sex, n (%)	
Male	1397 (62.1)
Female	852 (37.9)
Comorbidities, n (%)	
Any comorbidity	1997 (88.8)
Hypertension	1198 (53.3)
Coronary artery disease	487 (21.7)
Diabetes mellitus	234 (10.4)
Cerebrovascular disease	203 (9.0)
Malignancy	183 (8.1)
Bronchiectasis	103 (4.6)
Chronic kidney disease	103 (4.6)
Alzheimer’s disease	75 (3.3)
Diffuse interstitial lung disease	19 (0.8)
Primary admission diagnosis, n (%)	
COPD exacerbation	1024 (45.5)
Pneumonia	757 (33.7)
Asthma exacerbation	468 (20.8)

Note: COPD, chronic obstructive pulmonary disease; SD, standard deviation. Comorbidities were not mutually exclusive.

**Table 2:** Distribution of respiratory viral detections by primary admission diagnosis.

Viral Target	COPD Exacerbation (*n* = 1024)	Pneumonia (*n* = 757)	Asthma Exacerbation (*n* = 468)
**Influenza A**	**88 (8.6)**	**74 (9.8)**	**50 (10.7)**
A(H1N1)	60 (5.9)	48 (6.3)	31 (6.6)
A(H3N2)	65 (6.4)	43 (5.7)	35 (7.5)
**Influenza B**	**5 (0.5)**	**7 (0.9)**	**6 (1.3)**
**SARS-CoV-2**	**41 (4.0)**	**58 (7.7)**	**17 (3.6)**
**Other respiratory viruses**	**170 (16.6)**	**118 (15.6)**	**108 (23.1)**
**Adenovirus**	**10 (1.0)**	**10 (1.3)**	**8 (1.7)**
**Seasonal human coronavirus**	**51 (5.0)**	**33 (4.4)**	**26 (5.6)**
**Enterovirus/Rhinovirus**	**56 (5.5)**	**39 (5.2)**	**38 (8.1)**
**Human bocavirus**	**5 (0.5)**	**3 (0.4)**	**4 (0.9)**
**Human metapneumovirus**	**14 (1.4)**	**8 (1.1)**	**10 (2.1)**
**Human parainfluenza virus 1–4**	**16 (1.6)**	**3 (0.4)**	**9 (1.9)**
**RSV A/B**	**35 (3.4)**	**25 (3.3)**	**22 (4.7)**
**Parechovirus**	**1 (0.1)**	**0 (0.0)**	**0 (0.0)**

Note: Data are n (%). RSV, respiratory syncytial virus. “Other respiratory viruses” comprised all respiratory viruses included in the multiplex PCR panel other than Influenza A, Influenza B, and SARS-CoV-2. Virus-specific categories were not mutually exclusive.

**Table 3:** Distribution of respiratory viral detections by study period.

Viral Target	Period 1 *n* = 424	Period 2 *n* = 911	Period 3 *n* = 914	*p*-Value
**Influenza A**	**35 (8.3)**	**115 (12.6)**	**62 (6.8)**	**<0.001**
A(H1N1)	25 (5.9)	100 (11.0)	14 (1.5)	<0.001
A(H3N2)	33 (7.8)	66 (7.2)	44 (4.8)	0.104
**Influenza B**	**1 (0.2)**	**9 (1.0)**	**8 (0.9)**	**0.457**
**SARS-CoV-2**	**18 (4.2)**	**84 (9.2)**	**14 (1.5)**	**<0.001**
**Other respiratory viruses**	**68 (16.0)**	**154 (16.9)**	**174 (19.1)**	**0.308**
**Adenovirus**	**2 (0.5)**	**8 (0.9)**	**18 (2.0)**	**0.076**
**Seasonal human coronavirus**	**22 (5.2)**	**39 (4.3)**	**49 (5.4)**	**0.537**
*Coronavirus HKU1*	4 (0.9)	0 (0.0)	3 (0.3)	**0.016**
*Coronavirus 229E*	6 (1.4)	0 (0.0)	7 (0.8)	**0.004**
*Coronavirus NL63*	1 (0.2)	3 (0.3)	5 (0.5)	0.636
*Coronavirus OC43*	12 (2.8)	37 (4.1)	37 (4.1)	0.495
**Enterovirus/Rhinovirus**	**27 (6.4)**	**53 (5.8)**	**53 (5.8)**	**0.908**
**Human bocavirus**	**1 (0.2)**	**2 (0.2)**	**9 (1.0)**	**0.052**
**Human metapneumovirus**	**8 (1.9)**	**3 (0.3)**	**21 (2.3)**	**0.001**
**Human parainfluenza virus 1–4**	**11 (2.6)**	**9 (1.0)**	**8 (0.9)**	**0.020**
*Parainfluenza virus 1*	0 (0.0)	1 (0.1)	1 (0.1)	0.792
*Parainfluenza virus 2*	4 (0.9)	0 (0.0)	2 (0.2)	**0.007**
*Parainfluenza virus 3*	1 (0.2)	7 (0.8)	5 (0.6)	0.485
*Parainfluenza virus 4*	6 (1.4)	1 (0.1)	3 (0.3)	**0.003**
**RSV A/B**	**2 (0.5)**	**45 (4.9)**	**35 (3.8)**	**<0.001**
**Parechovirus**	**0 (0.0)**	**0 (0.0)**	**1 (0.1)**	**0.480**

Note: Data are n (%). Pearson’s chi-square test or Fisher’s exact test was used as appropriate. Bold *p*-values indicate statistically significant overall differences across the three study periods (*p* < 0.05). For variables with a significant overall difference, pairwise post-hoc comparisons were performed with Bonferroni correction for multiple comparisons. Significant pairwise differences were observed as follows: Influenza A, Period 2 vs. Period 3; A(H1N1), Period 1 vs. Period 2, Period 1 vs. Period 3, and Period 2 vs. Period 3; SARS-CoV-2, Period 1 vs. Period 2, Period 1 vs. Period 3, and Period 2 vs. Period 3; Coronavirus HKU1, Period 1 vs. Period 2; Coronavirus 229E, Period 1 vs. Period 2 and Period 2 vs. Period 3; human metapneumovirus, Period 1 vs. Period 2 and Period 2 vs. Period 3; human parainfluenza virus 1–4, Period 1 vs. Period 3; parainfluenza virus 2, Period 1 vs. Period 2; parainfluenza virus 4, Period 1 vs. Period 2; and RSV A/B, Period 1 vs. Period 2 and Period 1 vs. Period 3. Adjusted *p* < 0.05 was considered statistically significant for pairwise comparisons.

**Table 4:** Viral co-detection by study period.

Study Period	Total Patients, n	Viral Co-Detection, n (%)
Period 1	424	11 (2.6)
Period 2	911	56 (6.1)
Period 3	914	26 (2.8)
Total	2249	93 (4.1)

Note: Pearson’s chi-square test, *p* < 0.001.

**Table 5:** Viral combinations among patients with viral co-detection according to study period (*n* = 93).

Viral Combination	Period 1 n	Period 2 n	Period 3 n	Total n
Enterovirus + Rhinovirus	0	12	6	18
Influenza A + Influenza B	1	9	6	16
Influenza A + SARS-CoV-2	0	10	0	10
Coronavirus OC43 + Rhinovirus	0	3	2	5
Influenza A + Coronavirus OC43	1	3	0	4
Rhinovirus + Parainfluenza virus 4	3	0	0	3
SARS-CoV-2 + RSV	0	3	0	3
Adenovirus + human metapneumovirus	0	0	2	2
Enterovirus + human metapneumovirus	0	0	2	2
Influenza A + Adenovirus	0	2	0	2
Influenza A + Coronavirus 229E	1	0	1	2
Influenza A + RSV	0	2	0	2
Other two-virus combinations *	4	5	3	12
≥3 viruses detected	1	7	4	12
Total	11	56	26	93

Note: Each patient was counted once according to the complete viral detection pattern. Patients with three or more detected viruses were grouped as “≥3 viruses detected.” * Other two-virus combinations included combinations observed in only one patient. Period 1, September 2021–May 2022; Period 2, September 2022–May 2023; Period 3, September 2025–May 2026. RSV, respiratory syncytial virus.

## Data Availability

The de-identified data supporting the findings of this study are available from the corresponding author on reasonable request, subject to approval by the relevant institutional authorities and applicable privacy and ethical restrictions.

## References

[1] GBD 2021 Lower Respiratory Infections and Antimicrobial Resistance Collaborators (2024). Global, regional, and national incidence and mortality burden of non-COVID-19 lower respiratory infections and aetiologies, 1990–2021: A systematic analysis from the Global Burden of Disease Study 2021. Lancet Infect. Dis..

[2] Kahraman H., Keser G., Ölmezoğlu F.S., Altıntaş Öner B., Kurt O.S., Us T., Erdem F. (2025). Comparative clinical outcomes of major respiratory viruses in hospitalized adults during the post-pandemic period: A retrospective cohort study. Viruses.

[3] Dhanasekaran V., Sullivan S., Edwards K.M., Xie R., Khvorov A., Valkenburg S.A., Cowling B.J., Barr I.G. (2022). Human seasonal influenza under COVID-19 and the potential consequences of influenza lineage elimination. Nat. Commun..

[4] Huang Q.S., Wood T., Jelley L., Jennings T., Jefferies S., Daniells K., Nesdale A., Dowell T., Turner N., Campbell-Stokes P. (2021). Impact of the COVID-19 nonpharmaceutical interventions on influenza and other respiratory viral infections in New Zealand. Nat. Commun..

[5] Lei H., Xu M., Wang X., Xie Y., Du X., Chen T., Yang L., Wang D., Shu Y. (2020). Nonpharmaceutical interventions used to control COVID-19 reduced seasonal influenza transmission in China. J. Infect. Dis..

[6] Feng L., Zhang T., Wang Q., Xie Y., Peng Z., Zheng J., Qin Y., Zhang M., Lai S., Wang D. (2021). Impact of COVID-19 outbreaks and interventions on influenza in China and the United States. Nat. Commun..

[7] Achangwa C., Park H., Ryu S., Lee M.S. (2022). Collateral impact of public health and social measures on respiratory virus activity during the COVID-19 pandemic 2020–2021. Viruses.

[8] Zhao C., Zhang T., Guo L., Sun S., Miao Y., Yung C.F., Tomlinson J., Stolyarov K., Shchomak Z., Poovorawan Y. (2025). Characterising the asynchronous resurgence of common respiratory viruses following the COVID-19 pandemic. Nat. Commun..

[9] Korsun N., Trifonova I., Pavlova D., Grigorova I., Uzunova Y., Ivanov I., Ivanov D., Velikov P., Voleva S., Christova I. (2026). Viral pathogens in the etiology of acute respiratory infections in Bulgaria during the 2024–2025 season and genetic diversity of circulating influenza viruses. Front. Microbiol..

[10] Quarg C., Jörres R.A., Engelhardt S., Alter P., Budweiser S. (2023). Characteristics and outcomes of patients hospitalized for infection with influenza, SARS-CoV-2 or respiratory syncytial virus in the season 2022/2023 in a large German primary care centre. Eur. J. Med. Res..

[11] Vega-Piris L., Carretero S.G., Mayordomo J.L., Zarzuelo M.B.R., Río V.Á., García V.G., Vázquez M.G., Rodríguez M.D.C.G., Basile L., González-Coviella N.L. (2024). Severity of respiratory syncytial virus compared with SARS-CoV-2 and influenza among hospitalised adults ≥65 years. J. Infect..

[12] Newell K., Vroom N., Marques C., Maslova E., Khan F.L., Ngew K.Y., Volkman H.R., Wang C., Hernandez-Villena J.V., Aliabadi N. (2026). Viral pathogen-specific severe acute respiratory infections in hospitalised adults in Europe: Epidemiological findings from the id.DRIVE study 2024–2025. Infect. Dis. Ther..

[13] Xiong W., Cowling B.J., Tsang T.K. (2023). Influenza resurgence after relaxation of public health and social measures, Hong Kong, 2023. Emerg. Infect. Dis..

[14] Kim J., Kim Y.K., Song J., Kim D., Lee H. (2026). Influenza sentinel surveillance in the post-coronavirus disease 2019 period. Public Health Wkly. Rep..

[15] Havers F.P., Whitaker M., Melgar M., Pham H., Chai S.J., Austin E., Meek J., Openo K.P., Ryan P.A., Brown C. (2024). Burden of respiratory syncytial virus-associated hospitalizations in US adults, October 2016 to September 2023. JAMA Netw. Open.

[16] Silk B.J., Prill M.M., Winn A.K., Patton M.E., Moline H.L., Melgar M., Ma K.C., Paden C.R., Atherton L.J., Khan D. (2026). Respiratory virus activity—United States, July 1, 2024–June 30, 2025. MMWR Morb. Mortal. Wkly. Rep..

[17] Koçer İ., Demirbakan H., Aktaş A. (2025). Temporal dynamics and forecasting of respiratory viral infections during and after the SARS-CoV-2 pandemic (2020–2027): A multiplex PCR and ARIMA-based study. Front. Microbiol..

[18] Trifonova I., Korsun N., Levterova V., Pavlova D., Simeonovski I., Ivanova M., Velikov P., Voleva S., Ivanov I., Ivanov D. (2025). Respiratory infections in the post-COVID-19 era: Impact, prevalence, and clinical characteristics of bacterial and viral co-infections. Front. Med..

